# Susceptibility to Moral Arguments Among Liberals and Conservatives

**DOI:** 10.1093/poq/nfaf045

**Published:** 2025-11-12

**Authors:** Fredrik Jansson, Pontus Strimling

**Affiliations:** Associate Professor, Division of Mathematics and Physics, Mälardalen University, Västerås, Sweden; Guest Researcher, Centre for Cultural Evolution, Department of Psychology, Stockholm University, Sweden; and Affiliated Researcher, Institute for Futures Studies, Stockholm, Sweden; Associate Professor, Institute for Futures Studies, Stockholm, Sweden; and Adjunct Professor, Institute for Analytical Sociology, Linköping University, Norrköping, Sweden

## Abstract

An empirical result in Moral Foundations Theory is that liberals and progressives endorse the individualizing factors of care and fairness, while conservatives claim that the binding factors of authority, loyalty, and purity are equally relevant when determining what is moral. Does this translate into persuasiveness of arguments and opinion change? We here test the hypothesis that conservatives can be swayed by binding moral arguments, while everyone is susceptible to individualizing moral arguments. Using a classic experimental design (N = 375) where respondents are given moral arguments for a position in nine moral issues, we find support for this hypothesis. In line with motivational matching, the moral foundation support of respondents predicts the type of arguments to which they are susceptible. Along with previous studies on which type of moral argument supports which moral position in the public debate, these findings provide a mechanistic explanation for public opinion change, and in particular for the observation that moral values are becoming more liberal and progressive across the board. Although people tend to be resistant to belief revision, their opinions on politically polarized issues can change when arguments match their beliefs, reflected in their ideology.

## Introduction

People are polarized on a large number of moral and political issues, and opinions are often divided along party lines (e.g., Baldassarri and Gelman 2008). For example, in the United States, conservatives and liberals often take opposing views toward issues such as gay rights, gun regulation, abortion, and capital punishment (Treier and Hillygus 2009). However, for the same issues, public opinion has moved over time, and not in opposite directions, but rather consistently towards more liberal opinions on moral issues, with only a few exceptions (Mulligan, Grant, and Bennett 2013). This change is too rapid to be explained by generational value differences (e.g., the “settled dispositions” view; see Kiley and Vaisey 2020), but it seems that individuals, both liberals and conservatives, have adopted more liberal values over time (Strimling et al. 2019).

Meanwhile, numerous studies show how psychological biases preserve beliefs, and even in the face of counterevidence, the effect is often entrenchment, rather than taking on the opposing view (Nyhan and Reifler 2010; though see also Wood and Porter 2019; Guess and Coppock 2020). Taken together, trends in public opinion suggest that people do change their moral views, in the same direction, even though they tend to conserve their beliefs and reject arguments from the other side. The question is then what can actually cause people to change their minds on moral issues. There is a theoretical suggestion: conceptual and mathematical models of belief systems show that the degree of compatibility between different messages modulates rates of change in public opinion and can generate both conformism and attitude polarization (Jansson et al. 2021; Buskell, Enquist, and Jansson 2019). In line with this, on the empirical side, motivational matching (e.g., Joyal-Desmarais et al. 2022) is a strategy of aligning persuasive appeals with an individual’s underlying motives or values, which can effectively change people’s minds on moral issues by resonating with their core beliefs and values, thereby reducing resistance to change. Thus, if we could identify people’s core beliefs and values, then we may also be able to predict what types of messages can trigger opinion change and for whom.

Moral Foundations Theory (Haidt 2007) has identified some potential such core beliefs and values. The theory itself proposes that moral reasoning is mostly intuitive and based on a relatively small set of foundations. An important finding (Graham, Haidt, and Nosek 2009) is that both liberals and conservatives find the group of *individualizing* foundations relevant, which focus on the protection and fair treatment of individuals, emphasizing values of care, avoiding harm, and reciprocal fairness. Meanwhile, conservatives also endorse *binding* considerations, which stress group cohesion and social order, emphasizing values of group loyalty, respect for authority, and sanctity. Building on these results and the idea of motivational matching, we hypothesize that moral arguments based on the binding foundations will appeal to conservatives, and not liberals, with the ability to change conservative opinion, while both political groups will be open to arguments based on individualizing considerations, with the ability to change general public opinion. There is indirect support for this hypothesis, in that models and experiments that have *assumed* this to be the case have generated good predictions of public opinion change (Eriksson and Strimling 2015; Strimling et al. 2019).

There is some related work on moral reframing (for an overview, see Joyal-Desmarais et al. 2022), but only a few experimental studies that try to change respondents’ opinions by using arguments based on the moral foundations (Day et al. 2014; Feinberg and Willer 2015, 2019; see also Voelkel, Mernyk, and Willer 2023), with related but different research questions and testing an alternative hypothesis that individualizing arguments have an effect only on liberals. However, as mentioned above, surveys on moral foundation support have found that also conservatives endorse the binding foundations, and several studies connect that support to public opinion change at the population level (e.g., Clifford and Jerit 2013; Clifford et al. 2015; Miles 2016; Strimling et al. 2019; Strimling, Vartanova, and Eriksson 2022). Also, the moral reframing work in question has given mixed results, not clearly supporting either hypothesis and thus calling for further work. In this paper, we carry out an experiment designed to test our hypothesis directly. We will do so by asking liberal and conservative respondents for their opinions on nine moral issues before and after having been exposed to either type of moral arguments supporting the issues. If we find evidence that individuals do indeed change their opinion in the predicted direction, according to our hypothesis, then that will in turn provide a mechanistic explanation to public opinion change (Eriksson and Strimling 2015; Strimling et al. 2019), which in turn brings about specific predictions for it (Strimling, Vartanova, and Eriksson 2022).

## Background

We will here provide a brief background to public opinion change along partisan lines in the United States, the role and connections to individual belief updating, and, as our main focus, what factors can make individuals change their moral opinions. Based on the latter, we will formulate hypotheses for the kinds of arguments that will persuade liberals versus conservatives.

Polarization has long been a growing concern, not least in the United States, where people take on opposing views on moral issues along partisan lines (e.g., Treier and Hillygus 2009). However, while there has been increased party sorting, with moral views becoming more tightly connected to ideology, and increased polarization in elite rhetoric, there is less evidence for opinions becoming markedly more polarized in the general public (Fiorina and Abrams 2008; Hill and Tausanovitch 2015; DellaPosta 2020). In fact, liberals and conservatives have moved mostly in the same direction, towards typically “liberal” values, though some studies suggest that politically active voters have become more polarized (Evans 2003; Abramowitz and Saunders 2008). This pattern is, however, not inconsistent with temporarily increased polarization: when the population moves from one consensual state to another, for example as regards to the acceptance of homosexuality, the population becomes polarized during the transition, especially if one group moves faster than another (Fiorina and Abrams 2008). A remaining question, however, one which we will come back to, is what drives this commonly directed opinion change.

The broad prevalence of cognitive biases for conserving current worldviews may render it surprising that people change their opinions at all. *Belief perseverance*, the tendency to maintain a belief or attitude without supporting, or even in the face of contradicting, evidence, is a well-known phenomenon (Nisbett and Ross 1980) that is hard to counteract (Lewandowsky et al. 2012). Indeed, there is evidence that many dispositions are mostly settled at an early age (Kiley and Vaisey 2020). Theory on cognitive dissonance suggests that when hit by new information, people should avoid having to deal with opposing views (Nickerson 1998) and choose what provides the least psychological discomfort (Festinger 1957; Elliot and Devine 1994; Cooper 2007). People are more likely to accept information consistent with their present beliefs and values (Lewandowsky et al. 2012). Assessing compatibility is cognitively demanding, but people also use intuitive processing and affective responses, accepting a message that “feels right” (Lewandowsky et al. 2012).

A recent meta-study on matching effects (Joyal-Desmarais et al. 2022) found that it is possible to change attitudes, intentions and behaviors by matching messages to features of the audience, especially if messages are aligned with individual motivations, like goals, beliefs, and values. There are a few different approaches to message matching. Our approach would be closest to *motivational matching* to people’s beliefs and values. Here we also find related work on *moral reframing* (Feinberg and Willer 2019), for matching messages to differences in core moral values within political groups. While this approach appeals to reasoning, other potential strategies may target more affective responses. *Message framing* (which, despite the terminology, is different from moral reframing) targets obtained benefits or avoided costs from adopting focal behaviors, where motivational differences between individuals influence their responsiveness to gain versus loss. Within this area, regulatory focus theory specifically argues that people with a promotion or prevention focus are more influenced by potential gains or losses, respectively (e.g., Higgins 1998). Relatedly, when a message “feels right” from regulatory fit, it becomes more persuasive (Cesario, Grant, and Higgins 2004; Higgins 2005). As we will come back to in the discussion, this might improve the processing of moral arguments.

Moral Foundations Theory has identified a number of categories of values that seem to trigger intuitive responses and are widely used when people take positions for or against moral issues (Haidt 2007); they would thus seem instrumental for motivational matching. They also divide the US population along the liberal–conservative dimension (Haidt and Graham 2007; Graham, Haidt, and Nosek 2009): while conservatives claim that loyalty, authority, and purity—binding foundations—are relevant in moral considerations, liberals mostly do not. Liberals instead put a greater emphasis on care and fairness—individualizing foundations. It would seem, then, that moral considerations involving binding reasoning would target conservatives, while individualizing reasoning would target liberals, and this has been the focus of previous work on moral reframing, as we will describe below. On the other hand, this misses the finding that the individualizing foundations are in fact as relevant to the conservatives as the binding foundations. Somewhat simplified, this creates an important asymmetry, where some foundations appeal to everyone, and others only to conservatives. We will here investigate whether, beyond what people claim to value in moral considerations, this translates into a similar asymmetry in which types of arguments can change someone’s mind. Receiving an argument based on fundamental values you do have for a moral stance should impose a feeling of fit if you already agree with the stance, and some kind of dissonance if you did not agree with it. The argument may disrupt the sense of regulatory fit, and by providing compelling reasons the recipient believes in for the opposite stance, it should be easier to resolve this dissonance and achieve a stronger feeling of fit by changing your moral stance for the specific issue, to comply with your moral foundations, than it would be to change your moral foundations to comply with the stance.

One study (Day et al. 2014) used moral foundation language to reframe moral stances in each of the five foundations, in brief one-sentence formulations that varied more in phrasing than in content. This procedure moved both liberals and conservatives towards the liberal stances, but only conservatives towards the conservative stances, in line with other studies on public opinion change (Strimling et al. 2019). However, liberals were open to both individualizing and binding framings for the liberal stances, at odds with the lack of binding moral considerations in this group. The results thus suggest that verbal framings alone do not trigger responses in line with empirical moral foundation support.

A series of studies used more elaborate arguments for moral reframing (see Feinberg and Willer 2019 for an overview), designed to test the hypothesis that liberals are perceptible only to individualizing arguments, and conservatives only to binding arguments, the latter at odds with findings that conservatives endorse all foundations roughly evenly (as the authors also acknowledge). The first studied four moral stances (Feinberg and Willer 2015), analyzed separately, with mixed findings. The results provided support for an effect of moral reframing, but we would argue that they were inconclusive as to whether conservatives were susceptible to individualizing arguments, in part due to the fact that the hypothesis that they would be was not considered in the experimental design.

Two related studies tried to decrease versus increase support for political candidates using moral reframing. One (Voelkel and Feinberg 2018) found that a loyalty argument against Trump reduced conservatives’ support more than a fairness argument, while the difference was nonsignificant for liberals. Similarly, a fairness argument against Clinton decreased liberals’ support more than a loyalty argument, while for conservatives there was no difference. In another study, binding rhetoric among politically progressive candidates (Voelkel, Mernyk, and Willer 2023) increased conservative support, though without decreasing liberal support. Binding frames were thus generally successful, possibly because of novelty and reduced outgroup signaling. Again, these studies provide support for moral reframing, but further research is needed to investigate the effect of individualizing versus binding arguments.

Looking at the population level, however, there is indirect evidence that individualizing arguments have a universal impact, while binding arguments appeal only to conservatives, in line with moral foundation support in these groups. One study (Strimling et al. 2019) found that the net support for or against a moral issue by individualizing arguments explained more than half of the variation in the rate of public opinion change over 44 years for 74 moral issues, while binding arguments carried little explanative value. Individualizing argument support thus seems unique in explaining public opinion change in both groups. In line with this, other analyses at the aggregate found that elite rhetoric based on the care foundation works on those who support this foundation, and that such rhetoric is common, probably since the foundation is commonly endorsed (Clifford and Jerit 2013; Clifford et al. 2015; Miles 2016). It remains to test whether the mechanism suggested to lead to these outcomes indeed describes actual individual behavior.

Against this background, we hypothesize that it is possible to change people’s moral opinions by appealing to their moral foundations, and that there is an asymmetry in the general persuasiveness of arguments:

H: Both liberals and conservatives are susceptible to arguments based on the individualizing foundations, while only conservatives are susceptible to arguments based on the binding foundations.

This overarching hypothesis can be broken up into the following parts:

H1: Individualizing arguments will increase mean agreement relative to baseline agreement …

- for conservatives
- for liberals
- for the entire sample

H2: Binding arguments …

- will increase mean agreement relative to baseline agreement …
  - for conservatives
  - more so the more conservative the respondent is
- will have no discernible effect on liberals relative to baseline agreement
  - will have a larger effect on conservatives than liberals
  - will have a smaller effect on liberals than individualizing arguments have
    Note that H2b together with H2a implies H2b(i), and H2b together with H1b implies H2b(ii). We have included H2b(i) and H2b(ii) as a proxy for H2b since they are the alternatives of testable null hypotheses. Furthermore, since the predicted effects are suggested to be mediated by moral foundation support, such support should itself correspond to susceptibility to arguments:

H3: Moral arguments will increase mean agreement relative to baseline agreement when controlling for political ideology such that the effect is positively associated with …

- individualizing score among those exposed to individualizing arguments
- binding score among those exposed to binding arguments

## Methods

Given the mixed findings of previous studies, we have designed an experiment to specifically test our hypothesis. Our treatments go beyond verbal reframing, and since measured effects were expected to be small and noisy (as in the related studies, we should not expect people to radically change their moral opinions with only a brief argument), we used more issues, all with the same control–treatment design to allow a composite measure. We included a baseline control treatment, and, also contrasting to the previous studies, we measured actual opinion change at the individual level using a repeated measures design. This design enables direct comparisons between susceptibility to individualizing, binding, and no arguments, dependent on the recipients' morality and ideology.

Nine moral issues and stances were selected for their ability to provide convincing arguments based on both individualizing and binding foundations, and such that the individual foundations would all be represented roughly evenly. The arguments for the first six stances were loosely based on real arguments found in opinion pieces in newspapers and on discussion forums. The stances were against pornography, extramarital affairs, hate speech, suicide, and police violence, and for expanding governmental reach, universal healthcare, military spending, and same-sex marriage. The last three have been used in a previous study (Feinberg and Willer 2015), but then in a different setting and with more elaborate arguments. We tried to keep the arguments short and concise, so that the respondents would read them through and to avoid the risk of including inadvertent signals beyond the moral foundation in question that could influence the response. We also matched the length of the individualizing and the binding argument (average difference 9 percent). See table 1 for the moral stances and arguments.

**Table 1.** Moral stance items along with argument treatments.

Claim	
Binding argument	Individualizing argument
Pornography should be illegal.	
Pornography creates a perverted view of sexuality. It is indecent to those involved and promotes sinful behavior. Sex should be a special experience with a significant other.	The pornography business exploits women and puts them at risk of being abused. A ban would protect women from getting involved in harmful behavior.
It is always wrong for a married person to be unfaithful.	
A marriage is a devoted union where you stand together and show loyalty. By having an affair, you break a commitment and betray your closest ally.	Extramarital affairs harm your spouse. They impair his or her self-confidence, increase their fear of abandonment, and hurt them.
If a person wanted to make a speech in your community claiming that Black people are genetically inferior, then he should be banned from speaking.	
Allowing for extremist views to spread may threaten the stability of society. They can lead to subversive reactions and protests that defy social order.	Such speech is unjust and questions equal rights between people. It would spread intolerance, discrimination, and increase social exclusion.
A person has no right to end his or her own life, even if this person is tired of living and ready to die.	
Life is sacred, whether it is someone else’s or your own. Suicide is self-murder, so anyone who commits it is sinning, and disrespecting life.	Taking your life deeply hurts the people that care for you. Your close ones will suffer from grief and might feel that they are to blame.
The government should expand its reach to solve more of this country’s problems, rather than leaving them up to individuals and private businesses.	
There are many problems with disturbances and social unrest that we cannot come to terms with just between individuals. Authorities should be given more means to maintain order, and more governmental control is needed to reduce alienation. We need to stand together as a country.	There is still a lot of inequality in society, and problems with unfairness, discrimination, and segregation that we cannot come to terms with just between individuals. More governmental interventions are needed to make sure that people can have equal opportunities and equal rights.
I would disapprove of a policeman striking a citizen who was being questioned as a suspect in a murder case.	
The policeman needs to respect that it is the court’s decision whether to punish the citizen. No one may defy or disrespect the law by taking it in his own hands.	The police should protect, not harm, the citizens, criminals or not. Also, everyone has the right to equal treatment before the law.
I am in favor of universal healthcare.	
Universal healthcare is a way of purifying America from some of its most infectious diseases, spread by those who are uninsured. It will keep the country strong, and able to stand against our enemies.	Healthcare in the US is inherently unfair and unjust. Everyone, not just the rich and the fortunate, should have access to the doctors and the medicine they need.
Cutting funding to the military would be a mistake.	
The United States is the strongest nation in the world. Patriotic Americans have banded together in defense of what we hold dear and know is right. America must maintain its superpower status in the eyes of the world. It is our military strength that deters rogue states and terrorists from attacking us. It is also what unites us together at home in our most trying times.	The military helps defend human rights around the world. It helps to protect freedom of speech, association, and movement, and maintains democratic values. It ensures that also those who are not well off in society are protected, whether in the US or abroad, and not only the rich and empowered who have the means to fight for their own interests and rights.
I think marriage should be legal for same-sex couples.	
It is better for society if more people are married and avoid having relationships outside marriage. Stable relationships are the foundation of a stable society, and children need the stability that marriage can provide.	Preventing marriage among homosexuals is a direct form of discrimination. It is a message that we are not all equal, and also, a citizen cannot achieve true legal equality without the right to marry whomever they want.

The arguments have been constructed to correspond to the descriptions of the individualizing versus binding moral foundations (Graham, Haidt, and Nosek 2009; moralfoundations.org; e.g., Graham et al. 2011). The arguments also correspond to the Linguistic Inquiry and Word Count dictionary of moral foundations (Graham, Haidt, and Nosek 2009). A raw count of the words found both there and in these arguments gives that 88 percent are binding terms in our binding arguments, and 87 percent are individualizing terms in our individualizing arguments. Some individualizing terms can occur descriptively in binding arguments and vice versa due to the nature of the moral issue (e.g., the word “defense” for the military funding issue). As a further validation, we asked ChatGPT (GPT-4) to label each moral argument according to which moral foundation it uses (with the only pre-prompting being to ask for definitions of the moral foundations to verify that it represented them correctly). It labeled all of them as intended (see Supplementary Material section A).

We used a classic experimental (randomized control-group pretest post-test) design. The study was conducted on Amazon Mechanical Turk (MTurk), described only as “Give your opinions on current issues (prescreening)” in the posting. We collected data from 200 participants (out of which 87 participated in the second survey described below) in February–March 2018 and 1,000 (379) participants in April–May 2018. All participants were from the United States and were screened for ideology in a brief survey that paid $0.05, to get a balanced distribution of liberal and conservative respondents. Since the pool of MTurk workers has a liberal skew, this meant that all conservative and moderate and, randomly, a proportion (56 percent) of liberal respondents were invited to take the full survey for a bonus of $0.30. Those who accepted were then asked for their agreement with the nine moral stances, on the single-item scale from -100 to 100. We used a fine-grained scale so that the respondents would not remember their answers. One week later, the respondents were invited by email with a link to a follow-up study on MTurk for which they were offered a reward of $0.60. In the study, they were randomly allotted to one of three conditions. In the control condition, they were again asked for their agreement with the stances. In the two treatment conditions, they were first presented with an argument for each stance before rating their agreement with the stance in question. In one treatment, they received individualizing arguments for all stances, and in the other binding arguments. Finally, they filled in a Moral Foundations Questionnaire (MFQ, Graham et al. 2011) from which a score (0–5) could be calculated of how important each of the five foundations is to each participant. We included two attention checks, as provided by the MFQ. The survey protocol, including the wording of all questions, can be found in Supplementary Material section G. After prescreening, 859 respondents were offered to participate in the first survey, out of which 795 (93 percent) accepted and 466 (54 percent) also completed the second survey, about a week later. We excluded those respondents who answered that it was relevant (slightly or above) when assessing the morality of an action whether someone was good at math or who did not (at least moderately) agree that it is better to do good than bad. After these attention checks, 375 respondents remained for our analysis.

We used condition, political ideology, and individualizing and binding moral foundation scores as independent variables and the average difference in opinion for the nine moral stances from the first to the second measurement as the dependent variable in a linear model, using ordinary least squares regression. Note that all arguments in the treatment conditions were in the direction of increasing agreement with the moral stance. This design provided a composite measure for opinion change for each treatment and respondent to detect overall effects and reduce noise from individual items. Controlling for item contributions interacting with treatment would require several magnitudes of more data, but we provide descriptive results in Supplementary Material section C.

Ideology used a scale measure from extremely to slightly liberal (-3 to -1) through moderate (0) to slightly to extremely conservative (1 to 3). We used both a dichotomized version of this variable, with liberals (-3 to -1) and conservatives (1 to 3) as the two factors, to address the main hypotheses H1a, H1b, H2a(i), H2b(i), and H2b(ii), and the full scale measure for H1c, H2a(ii), and for the relations to the MFQ in H3a and H3b. The individualizing score is the average of the scores for care and fairness, and the binding score of loyalty, authority, and purity.

## Results

Our final sample of 375 respondents had similar characteristics to both those who dropped out and those excluded concerning age, gender, education, income, and ideology (55 percent women; mean age 38 years, median 35 years; 39 percent liberals, among which 11 percent slightly and 11 percent extremely, 18 percent moderates, 43 percent conservatives, among which 18 percent slightly and 5 percent extremely) and their answers to the moral issues. More details are given in Supplementary Material section B. We also repeat our main analyses without excluding respondents based on attention checks in Supplementary Material section F. First, the respondents’ endorsements of the foundations with respect to the position on the political scale are in line with previous observations that liberals say they mainly consider care and fairness, while conservatives say they consider all five (see Supplementary Material section C  figure S1). The average scores for the individualizing versus binding foundations were 3.7 versus 1.8 for liberals, 3.5 versus 2.6 for moderates, and 3.2 versus 2.9 for conservatives (for each foundation there are three moral relevance items that require at least a 3 for the respondent to agree rather than disagree, and three moral judgments that require at least a 2 for the respondent to find it at least slightly relevant, so it could be argued that the threshold for accepting rather than rejecting a foundation is 2.5). To check for potential posttreatment bias (Montgomery, Nyhan, and Torres 2018) from filling in the MFQ last in the survey, we compared MFQ scores across the treatments, to see if the treatment had an effect on the variable. We found no significant differences in individualizing (F(2,364) = 1.2, *p* = 0.302, one-way ANOVA) or binding (F(2,367) = 0.13, *p* = 0.883) scores between the treatments. This applies also to the separate foundations (ranging from F = 0.23, *p* = 0.793, for loyalty, to F = 1.27, *p* = 0.280, for fairness).

**Figure 1.**
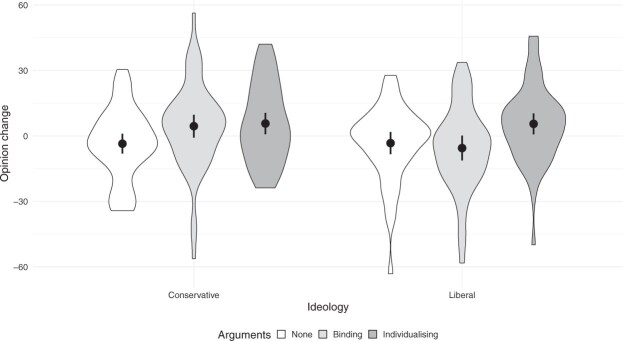
Distributions of opinion change, averaged over all nine issues, in the three conditions and by ideology. The central dots and lines represent means and 95 percent confidence intervals for the means.

Figure 1 shows the distributions over all conservative and liberal respondents of the average change in the nine moral issues from the first to the second measurement in the three conditions. The averages (and standard deviations) for conservatives are -3.5 (16.3), 4.5 (19.7), and 5.7 (17.7) for the control, binding, and individualizing conditions, respectively, while for liberals they are -3.3 (17.2), -5.5 (19.4), and 5.6 (17.5). As predicted (H1a, H2a(i)), the opinion change among conservatives is significant for both types of arguments (t(106.1) = 2.32, *p* = 0.022, Cohen’s d = 0.44, for binding and t(102.6) = 2.77, *p* = 0.007, d = 0.54, for individualizing arguments, Welch two sample two-sided t-tests), compared to the control condition, while for liberals (H1b, H2b), only individualizing arguments have a positive effect (t(95.5) = 2.53, *p* = 0.013, d = 0.51), significantly larger (t(93.2)= 2.98, *p* = 0.004, d = 0.60) than the effect from binding arguments (H2b(ii)). Opinion change is significantly larger (t(98.7) = 2.59, *p* = 0.011, d = 0.51) among conservatives receiving binding arguments than among liberals (H2b(i)). There is no significant difference where it was not hypothesized, between the binding and no argument conditions for liberals (H2b, t(90.1) = -0.59, *p* = 0.56, d = -0.12), and the individualizing and binding argument conditions for conservatives (t(107.9) = 0.34, *p* = 0.73, d = 0.06). The power of the tests ranges from 0.63 to 0.88 (see Supplementary Material section E for more details).

**Figure 2.**
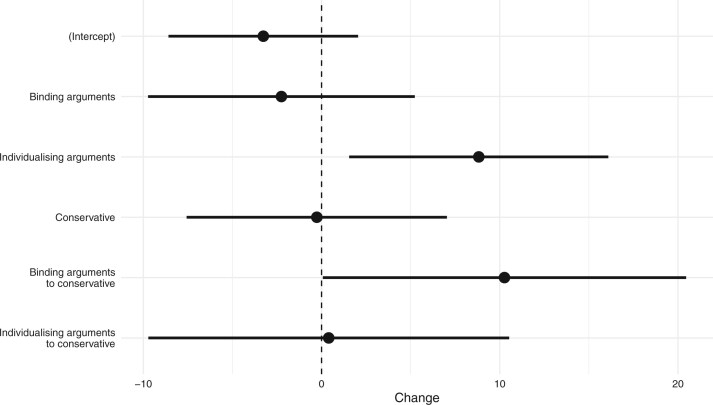
Estimated effects in terms of opinion change for each variable. The effect sizes are the regression coefficients, along with 95 percent confidence intervals, in the nominal ideology model.

In order to evaluate the effect of arguments and ideologies, against the control condition, both separately and in added interaction, figure 2 illustrates ordinary linear regression coefficients on the same data. The prediction is that individualizing arguments have an effect for both conservatives (H1a) and liberals (H1b), and that there is an interaction effect for receiving binding arguments and being conservative (H2a(i)). As predicted, there is no baseline difference in how much liberals and conservatives change, and binding arguments are nonpersuasive to liberals (H2b) but sway conservatives (β = 10.3, t(302) = 2.01, *p* = 0.045), while individualizing arguments have a general significant effect that is not connected to ideology (β = 8.8, t(302) = 2.43, *p* = 0.016).

In Supplementary Material section C, we list descriptive data on initial opinions and average change for each moral stance item, within each ideological group and condition. While too noisy for drawing conclusions at the item level, we note that most of the measured effects align with our main hypothesis, though the binding arguments for three items stand out for having either a positive or a negative measured effect for both liberals and conservatives. These exceptions support neither our hypothesis nor the alternative hypothesis described in the related work section, and excluding them from the analysis makes the hypothesized effects even more significant, and increases the model fit (adjusted R-squared increased from 0.050 to 0.071). See Supplementary Material section C for more details.

For a more fine-grained analysis, addressing the generalized prediction that degree of conservatism matters, we used the full-scale measure of ideology (including moderates). The descriptive data on initial opinions and average change in each political group and condition are presented in Supplementary Material section C. Table 2 lists linear regression results from two models. Including only arguments, disregarding ideology, individualizing arguments are overall significantly persuasive (H1c), while binding arguments are not. Using a scale measure of ideology gives a positive interaction effect between degree of being conservative and receiving binding arguments (H2a(ii)), and overall gives a similar result to the dichotomous one (except that binding arguments have a nonsignificant positive, instead of nonsignificant negative, estimated effect, which can be due to moderates being the reference group in this model). Including political ideology, in addition to argument type, increases the model fit significantly (F(3,369) = 3.78, *p* = 0.010). In Supplementary Material section D, we provide further analyses controlling for effects of initial opinions, showing that significant results remain.

**Table 2.** Linear models (OLS) of average change in opinion per respondent.

	Arguments	Ideology
(Intercept)	−2.43 ± 1.61	−2.42 ± 1.60
	(0.133)	(0.131)
Binding args	3.13 ± 2.31	3.07 ± 2.29
	(0.176)	(0.180)
Individualizing args	7.62 ± 2.28	7.56 ± 2.27
	(<0.001)	(<0.001)
Conservative		−0.22 ± 0.90
		(0.806)
Binding args × conservative		3.23 ± 1.27
		(0.011)
Individualizing args × conservative		−0.00
		(0.999)
*N*	375	375
F-statistic	5.63	4.57
	(0.004)	(<0.001)
Adjusted R^2^	0.024	0.046

Unstandardized coefficients are listed with standard errors, with *p*-values below in parentheses. Ideology uses a scale measure from extremely to slightly liberal (-3 to -1) through moderate (0) to slightly to extremely conservative (1 to 3). The independent variables are type of argument received (with no argument as a reference), being conservative (rather than liberal), and interactions (denoted by ×) between type of argument and ideology.

The rationale for our hypothesis was that liberals endorse only the individualizing foundations, while conservatives find all five to be of relevance. If this is the explanation, then we would expect the results from the MFQ to be associated with opinion change also when controlling for political ideology (H3a, H3b). Since the MFQ was given posttreatment, we analyze the treatment groups separately, to guarantee that the samples remain balanced and unbiased (i.e., all respondents in each analysis have received the same treatment). Table 3 shows linear regression results in the three groups. As expected, measured effects are close to zero and nonsignificant in the control group. In all groups, political ideology is nonsignificant when including MFQ scores. In the group that received binding arguments, opinion change is negatively associated with individualizing score, for which case there was no prediction.

**Table 3.** Linear models (OLS) of average change in opinion per respondent in the three conditions (type of argument received).

	Control	Binding	Individualizing
(Intercept)	0.56 ± 7.82	10.23 ± 10.48	−38.26 ± 8.86
	(0.943)	(0.331)	(<0.001)
Conservative	−0.06 ± 1.11	−0.35 ± 1.46	1.08 ± 1.24
	(0.954)	(0.809)	(0.387)
Binding moral	−0.52 ± 2.04	6.92 ± 2.73	2.31 ± 2.11
	(0.797)	(0.013)	(0.277)
Individualizing moral	−0.53 ± 2.30	−7.77 ± 3.24	10.70 ± 2.39
	(0.818)	(0.018)	(<0.001)
*N*	125	113	125
F-statistic	0.07	5.58	8.46
	(0.976)	(0.001)	(<0.001)
Adjusted R^2^	−0.002	0.109	

Unstandarized coefficients are listed with standard errors, with *p*-values below in parentheses. Ideology uses a scale measure from extremely to slightly liberal (-3 to -1) through moderate (0) to slightly to extremely conservative (1 to 3). The independent variables are being conservative and moral foundation scores.

The other two significant effects across the three analyses are the predicted ones. In the binding treatment group, opinion change is positively associated with binding foundation support (H3b), and among those receiving individualizing arguments, that association holds for individualizing foundation support (H3a).

We provide an analysis of the whole sample and interactions between type of arguments and moral foundation support in Supplementary Material section D, to allow for comparisons between the models. Indeed, the model fit increases significantly (F = 5.42, *p* < 0.001). In that model, however, the sample is not guaranteed to be balanced and unbiased with respect to confounders, but at least we find no indications of posttreatment effects (difference between treatments in individualizing scores: F(2,364) = 1.2, *p* = 0.302; binding scores: F(2,367) = 0.13, *p* = 0.883; one-way ANOVA).

We repeated our main analyses for all respondents, including those who were filtered out based on attention checks. The respondents in question provided less consistent responses and spent less time than the others, indicating that their answers are less reliable and contain more noise. The results are presented in Supplementary Material section F and are similar with respect to how ideology predicts opinion change across conditions. The associations with moral foundation scores are mostly similar, except for an unexpected positive association between opinion change and binding score in the individualizing argument treatment. The latter association is driven by respondents who answered that it is morally relevant whether someone is good at math. A possible explanation is that those respondents tend to answer higher on the scale across all items, and thus their responses have a greater impact on variables where other respondents tend to score low. The additional significant effect is thus at least consistent with and potentially explained by increased noise (around a different mean than for the respondents who passed the attention checks).

## Discussion

Arguing for nine moral stances, it was possible to convince the respondents to agree more, on average, with the stances, provided that the arguments were tailored to the respondents’ support for individualizing versus binding moral foundations. In terms of political ideology, this meant that conservatives were swayed by arguments based on any of the moral foundations, while liberals were influenced only by individualizing arguments. As was discussed earlier, this finding contrasts with the hypothesis of previous work within the “moral reframing” literature (Feinberg and Willer 2019) in that conservatives are susceptible also to individualizing arguments, but it is consistent with the relative moral foundation support among liberals and conservatives that has been reported earlier (Graham, Haidt, and Nosek 2009) and that we find also in our sample (see figure S1), and with previous findings on public opinion moving in the direction of the stance with the greatest individualizing support (Miles 2016; Strimling et al. 2019). The average opinion change for those receiving the right kind of argument was about five points, which is small relative to the scale, but it should be recalled that changing people’s opinions on individual moral issues is hard, and the respondents received only a few sentences. However, since it is a hard problem, and the effects in the experiment are consequently small (while significant), more research is called for, for example to test the hypothesis in various settings, and the persistence and internalization of the changed opinions (c.f. Jansson and Bursell 2018), beyond direct effects after being exposed to the stimulus.

As expected, the susceptibility to arguments in the respective political groups corresponded to moral foundation support, beyond ideology. We cannot from this conclude which variable imposes which: moral foundation support might lead people to adopt a political ideology, but the direction of causality could also be the reverse, that moral foundation support is caused by political ideology. A recent study found support for the latter (Hatemi, Crabtree, and Smith 2019), suggesting that moral evaluations are rooted in motivated reasoning anchored in political beliefs. The causal link between moral foundations and political ideology remains an open question, but a suggestion is that certain political ideas go well with certain moral foundations, and if political ideas are connected so that they, and the people who endorse them, form clusters, then moral foundation support might cluster similarly (c.f. Buskell, Enquist, and Jansson 2019; Jansson, Buskell, and Enquist 2023).

There are a few other notable limitations with the present study, some deriving from the challenge of persuasion through brief arguments, some calling for further research. While MTurk is generally a source of high-quality data and has a more diverse representation than many offline settings (Paolacci, Chandler, and Ipeirotis 2010; Casler, Bickel, and Hackett 2013), such as lab experiments at universities, it still has a demographic skew, and especially we call for replication beyond American respondents. Within the survey, the order of measures and treatments may incur priming effects. Here we tried to minimize priming before the treatment and to avoid demand characteristics (Orne 1962; Nichols and Maner 2008; though see Sheagley and Clifford 2025) by placing the MFQ at the end, with the potential instead for posttreatment bias (Montgomery, Nyhan, and Torres 2018). However, we found no indication that posttreatment variables differed at the group level between the treatments. Individual heterogeneity is still possible, so we analyzed the treatment groups separately.

We here designed the survey such that a composite measure could assess opinion change across items, as we are interested in average effects from moral foundation support. As explained in the introduction, persuasion is hard and opinion change rare, so we expected small effects. This is also confirmed by the previous single-item studies described in the related work section, with ambiguous and noisy results. Since changes on the item level are highly variable and noisy, studies that use single items run the risk of finding effects that cannot be clearly attributed to moral foundations. At the item level, there are also threshold effects and high variability in initial opinions, especially for individual respondents. The average standard deviation per item here is 48, while for the composite it is 18. The composite measure comes at the disadvantage of not identifying the relative contribution from each item, where potentially a few items could drive the results and others dilute the overall effect. We expect relative contributions to be small, not necessarily significantly nonzero, and thus sometimes pointing in the wrong direction. Descriptive data show no clear indications of a single item driving the results (see Supplementary Material section C), though three items show measures in unexpected directions (against both our hypothesis and the alternative one in related work). Those items did not drive the results, but on the contrary, excluding them increased the measured effects in line with our hypotheses. Ideally, a study with few resource constraints could make more rigorous constructions and validations of moral foundation powered arguments and collect multitudes more of data to control for item interactions. The current constraints make the test conservative for detecting the hypothesized effect, though they should not increase the risk of Type I errors.

Our hypothesis is based on motivational matching (Joyal-Desmarais et al. 2022) along with findings from studies on moral foundations (e.g., Graham, Haidt, and Nosek 2009). A potential explanation for how it works is that people are more susceptible to arguments that provide cognitive consonance between current values and the moral stance in question, that is, information that is consistent with what they already believe (e.g., Jansson et al. 2021; Jansson, Buskell, and Enquist 2023). This would not necessarily entail high-level cognitive processes, but arguments work when they fit or “feel right” (Lewandowsky et al. 2012; Kidwell, Farmer, and Hardesty 2013), here when they are tailored to the receiver’s moral foundation. Similar effects might be achieved with other persuasion frameworks, such as Regulatory Focus Theory (e.g., Higgins 2005), where creating regulatory fit is related to matching moral foundations to receivers. A prevention focus might have connections to the binding foundations and a promotion focus to the individualizing ones, and both regulatory fit and moral arguments might increase the value of advocated opinions by increasing value from fit and avoiding dissonance and discomfort from mismatched arguments.

There are other possible factors involved in personalized matching, apart from the fit of the message itself to your other ideas (Teeny et al. 2021). One source of potential dissonance is who delivers the message. People may be more likely to accept information from certain people over others (e.g., conservatives may be more likely to be persuaded by conservatives, described as a type of information filtering by Jansson et al. 2021). Using binding arguments may be important for conservative candidates when arguing for typically liberal stances, to signal that they are still part of the ingroup (Voelkel, Mernyk, and Willer 2023). Added to this effect, we are more exposed to people similar to us (e.g., McPherson, Smith-Lovin, and Cook 2001). Future work could investigate the relative effect of varying the sender of the arguments.

As described in the background section, apart from a bias for what type of arguments to accept, people also likely choose what type of arguments to share with others in concordance with their other beliefs. In a pilot study (N = 88), we found that liberals used a significantly higher proportion of individualizing arguments than conservatives, who used individualizing and binding arguments equally often, when asked to argue for ten moral stances. Thus, also when arguing for the same position, the arguments are based on different moral foundations. Feinberg and Willer (2015) also found that rather than considering the receiver of the message when trying to persuade someone, their respondents made arguments that were grounded in their own moral values. This suggests that people’s own fit determines the type of arguments they use rather than the moral values of the person they are trying to convince (see Jansson et al. 2021 for a formal model of such a process).

Taken together, this suggests that people use the same type of arguments as those they are persuaded by, which are arguments that fit with other values the person has. Here it means that liberals use individualizing arguments for their causes, while conservatives often also use binding arguments, and that everyone is susceptible to arguments made by liberals, while conservatives’ arguments will often appeal only to other conservatives. There is thus one type of ideas appealing only to certain clusters of people, while everyone is open to another type, resulting in the spread of the type of ideas held by the most restrictive individuals (Acerbi, Enquist, and Ghirlanda 2009). The prediction from these processes at the microscale is the macro-outcome that public opinion will tend towards liberal values (Eriksson and Strimling 2015), which has been confirmed to be the case (Mulligan, Grant, and Bennett 2013), and that individualizing argument support for a moral stance predicts the rate of opinion change towards that stance (Strimling et al. 2019). Our results provide evidence that the suggested mechanisms behind this public opinion change are as assumed.

## Supplementary Material

nfaf045_Supplementary_Data

## Data Availability

Replication data and documentation are available at https://doi.org/10.7910/DVN/OHBTUC.
