# Supplementary material for: Susceptibility to Moral Arguments Among Liberals and Conservatives
Source: Public Opin Q. 2025 Nov 12;89(3):735–57. doi: 10.1093/poq/nfaf045 (PMC12695052; doi:10.1093/poq/nfaf045)
Supplement: nfaf045_Supplementary_Data [file nfaf045_supplementary_data.pdf]

Susceptibility to Moral Arguments Among Liberals and Conservatives

Supplementary Material

Fredrik Jansson[1,2,3,*] and Pontus Strimling[3]

[1]*School of Education, Culture and Communication*
Mälardalen University
Box 883, SE-721 23 Västerås

[2]*Centre for Cultural Evolution*
Department of Psychology
Stockholm University
SE-106 91 Stockholm

[3]*Institute for Futures Studies*
Box 591, SE-101 31 Stockholm

[*]Correspondence to: fredrik.jansson@su.se

Contents

A. Moral stances and arguments 2
B. Respondents 3
C. Descriptive results 4
D. Linear models 6
E. Power analyses 7
F. Excluded respondents 7
G. Survey protocol 10

A. Moral stances and arguments

We asked ChatGPT (GPT-4) to label each moral argument according to which moral foundation it uses. Here is the full conversation (the omitted parts can be found at https://chatgpt.com/share/6723a65b-ddb8-8013-8e91-7684e2705a60):

USER: Define the five moral foundations of Moral Foundations Theory.

CHATGPT: Moral Foundations Theory, proposed by social psychologists Jonathan Haidt and Craig Joseph, suggests that people's moral reasoning is based on five fundamental moral foundations. [Appropriate definitions of the foundations in 2–3 sentences each.]

USER: I will give you a number of moral claims, along with two arguments for the claim. Your task is to label each argument according to which of the moral foundations it uses. [See Table 1 in the manuscript. The *a* arguments are from the Binding column and the *b* arguments from the Individualising column.]

CHATGPT: Here's how each argument aligns with the five moral foundations of Moral Foundations Theory:

Claim 1: Pornography should be illegal.

- Argument 1a: Sanctity/Degradation. This argument appeals to the sacredness of sex and the degradation involved in pornography.
- Argument 1b: Care/Harm. This argument focuses on protecting women from exploitation and harm.

Claim 2: It is always wrong for a married person to be unfaithful.

- Argument 2a: Loyalty/Betrayal. This argument emphasizes the betrayal involved in infidelity and the importance of loyalty in marriage.
- Argument 2b: Care/Harm. This argument is concerned with the emotional harm caused to the spouse by infidelity.

Claim 3: Banning a speech claiming racial inferiority.

- Argument 3a: Authority/Subversion. This argument involves maintaining social order and the potential for subversion of societal stability through extremist views.
- Argument 3b: Fairness/Cheating. This argument appeals to justice, equal rights, and the prevention of discrimination and social exclusion.

Claim 4: A person has no right to end his or her own life.

- Argument 4a: Sanctity/Degradation. This argument views life as sacred and considers suicide a form of self-degradation.
- Argument 4b: Care/Harm. This argument is about the emotional harm suicide causes to loved ones.

Claim 5: Government should solve more problems.

- Argument 5a: Authority/Subversion. This argument advocates for stronger government authority to ensure social order and unity.
- Argument 5b: Fairness/Cheating. This argument focuses on addressing inequality and ensuring fairness and equal rights through governmental intervention.

Claim 6: Disapproving of police violence.

- Argument 6a: Authority/Subversion. This argument insists on respect for the rule of law and the proper authority of the courts.
- Argument 6b: Care/Harm. This argument emphasizes the police's duty to protect all citizens and ensure equal treatment before the law.

Claim 7: In favor of universal health care.

- Argument 7a: Sanctity/Degradation. This argument concerns the purification of society from disease, viewing health as a sacred aspect that strengthens the nation.
- Argument 7b: Fairness/Cheating. This argument is about rectifying injustice and ensuring equitable access to healthcare for all.

Claim 8: Opposing cuts to military funding.

- Argument 8a: Loyalty/Betrayal. This argument highlights the importance of national unity, patriotism, and maintaining America's strength and deterrence capability.
- Argument 8b: Fairness/Cheating. This argument concerns the military's role in defending human rights and democratic values globally, ensuring protection for all, not just the privileged.

Claim 9: Legal marriage for same-sex couples.

- Argument 9a: Loyalty/Betrayal (with elements of Sanctity/Degradation considering the focus on societal stability and the sanctity of marriage).
- Argument 9b: Fairness/Cheating. This argument addresses the discrimination against homosexuals and advocates for legal and social equality, including the right to marry.

B. Respondents

After pre-screening 1,200 respondents residing in the US, 859 respondents were offered to participate in the first survey, out of which 795 (93%) accepted and 466 (54%) also completed the second survey, about a week later. There were no notable differences between the sample of respondents who completed the survey and those who dropped out concerning age, gender, education, income and ideology (53% vs. 55% women; mean age 37 vs. 36 years; 38% vs. 42% liberals and 42% vs. 36% conservatives, $\chi^2(2) = 4.4$, $p = .11$; almost identical education levels: $\chi^2(4) = 0.56$, p = .97; similar income distributions: $\chi^2(9) = 10.9$, $p = .28$; no significant difference in distributions along a 7-point ideology scale: $\chi^2(6) = 7.2$, $p = .30$), and their answers to the moral issues were similar (average difference in mean points per moral item 5 out of 200, with a higher rating equally often in either sample). We included two attention checks, as provided by the Moral Foundations Questionnaire (see also Feinberg and Willer 2015). We excluded those respondents who answered that it was relevant (slightly or above) when assessing the morality of an action whether someone was good at maths or who did not (at least moderately) agree that it is better to do good than bad. This left 375 respondents (55% women; mean age 38 years, median 35 years; 39% liberals, 18% moderates, 43% conservatives, with $\chi^2(2) = 2.6$, $p = .28$, compared to excluded respondents), for which our analysis will pertain.

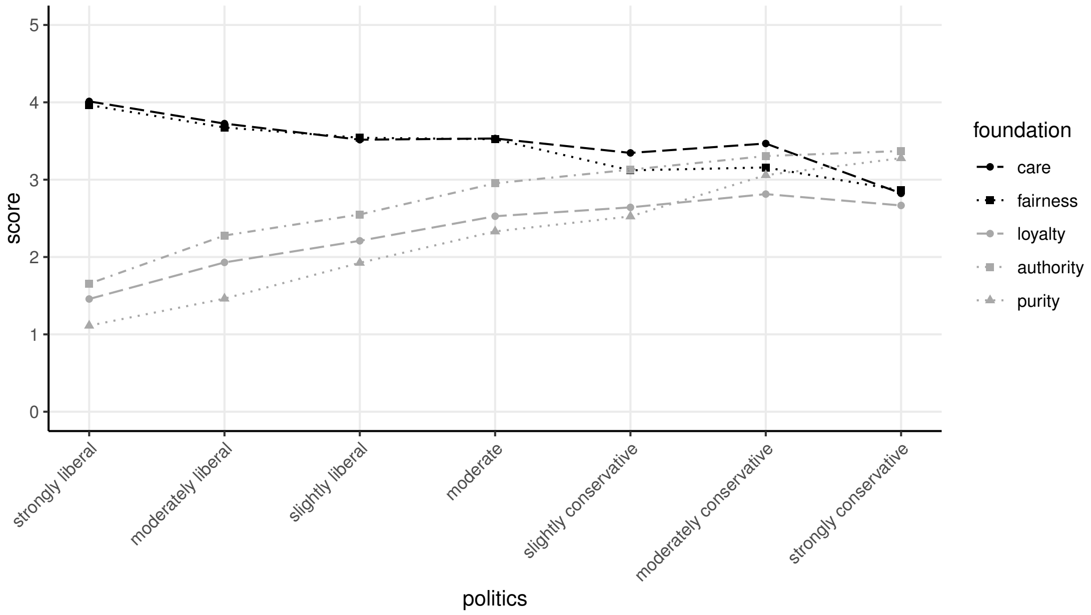

Figure S1: Moral relevance and judgements of the five foundations with respect to political ideology.

	Control			Binding			Individualising		
Strongly L	30 ± 8	−6 ± 17	(10)	17 ± 14	−16 ± 21	(13)	21 ± 17	7 ± 12	(20)
Moderately L	27 ± 17	−1 ± 14	(24)	19 ± 19	−7 ± 18	(21)	15 ± 16	10 ± 17	(18)
Slightly L	14 ± 16	−5 ± 23	(12)	3 ± 18	7 ± 13	(13)	16 ± 17	−1 ± 23	(15)
Moderate	18 ± 27	1 ± 18	(29)	10 ± 19	5 ± 17	(17)	14 ± 27	3 ± 20	(21)
Slightly C	9 ± 14	−1 ± 13	(15)	7 ± 18	6 ± 18	(25)	5 ± 19	3 ± 18	(27)
Moderately C	2 ± 29	−4 ± 17	(29)	6 ± 25	2 ± 19	(25)	5 ± 18	10 ± 17	(23)
Strongly C	4 ± 29	−7 ± 21	(8)	5 ± 30	6 ± 29	(7)	5 ± 13	−7 ± 15	(3)

Table S1: Descriptive data on average initial opinion, average change and number of respondents (in brackets) for each political category and condition. Numbers after ± are standard deviations.

C. Descriptive results

The scores in the Moral Foundation Questionnaire for each foundation and political category are presented in Figure S1. The initial average opinions and average change are presented in Table S1 divided into the seven political categories and the three conditions.

Table S2 presents the same measures but within the broader categories of conservatives and liberals for each moral stance. The standard deviations are relatively high on the item level, and thus it is hard to draw any strong conclusions at this level of analysis. Most items have an estimated effect in the direction of the hypothesis (positive for conservatives in the binding

		Control		Binding		Individualising	
1. Pornography (−)	C	−22 ± 64	0 ± 31	−21 ± 67	10 ± 47	−39 ± 62	18 ± 59
	L	−59 ± 47	3 ± 38	−71 ± 40	6 ± 35	−61 ± 51	6 ± 40
2. Extramarital affairs (−)	C	55 ± 47	−2 ± 30	55 ± 51	8 ± 48	67 ± 42	−2 ± 40
	L	33 ± 45	2 ± 42	28 ± 51	18 ± 50	31 ± 51	5 ± 42
3. Hate speech (−)	C	−14 ± 62	−11 ± 40	−12 ± 61	3 ± 70	−36 ± 63	3 ± 49
	L	15 ± 60	−4 ± 38	1 ± 58	−7 ± 45	3 ± 63	2 ± 56
4. Suicide (−)	C	−5 ± 63	−1 ± 72	−19 ± 57	17 ± 61	−15 ± 69	10 ± 69
	L	−25 ± 53	−6 ± 69	−51 ± 56	−3 ± 50	−54 ± 57	24 ± 58
5. Governmental reach (+)	C	−37 ± 51	−3 ± 41	−27 ± 57	7 ± 45	−26 ± 53	−6 ± 50
	L	37 ± 40	−12 ± 44	28 ± 48	−22 ± 53	38 ± 48	5 ± 48
6. Police violence (−)	C	35 ± 52	−21 ± 62	38 ± 50	12 ± 58	37 ± 56	14 ± 46
	L	68 ± 38	0 ± 43	58 ± 43	2 ± 41	67 ± 40	−4 ± 43
7. Universal health care (+)	C	−20 ± 56	7 ± 36	6 ± 64	−16 ± 45	−11 ± 60	10 ± 52
	L	76 ± 33	3 ± 23	69 ± 42	−21 ± 50	73 ± 33	−1 ± 35
8. Military spending (+)	C	47 ± 48	1 ± 41	38 ± 55	3 ± 52	50 ± 51	−1 ± 40
	L	−9 ± 46	−11 ± 45	−26 ± 50	2 ± 38	−26 ± 44	17 ± 39
9. Same-sex marriage (+)	C	−2 ± 73	−2 ± 38	1 ± 65	−3 ± 54	15 ± 74	5 ± 51
	L	80 ± 45	−4 ± 41	88 ± 24	−26 ± 62	84 ± 33	−4 ± 22

Table S2: Descriptive data on average initial opinion and average change for each item (with + or − indicating that the claim is for or against, respectively), political category (C for conservatives, L for liberals) and condition. Numbers after ± are standard deviations. The number of respondents is 52, 57 and 53 conservative, and 46, 47 and 53 liberal respondent in the no, binding and individualising argument condition, respectively.

condition, positive for both groups in the individualising condition). For some items, we likely see threshold effects: conservatives in the individualising condition in item 2, liberals in the same condition in item 6, and liberals in items 7 and 9 all have positive initial opinions above two thirds of the maximum. Three binding arguments stand out: in item 2, it is measured as positive also for liberals, and in items 7 and 9, it is measured as negative for both liberals and conservatives. This is neither in line with our hypothesis, nor the alternative one that liberals and conservatives are susceptible to exclusively individualising or binding arguments, respectively. We would thus take this as evidence for neither, but conclude that the binding arguments for items 7 and 9 are in themselves not very persuasive, while for item 2, it potentially taps into the care/harm foundation, thus appealing to both groups. Note that the inclusion of these items are expected to dilute the aggregated effect and thus making the test more conservative in terms of rejecting the null hypothesis and providing evidence for our hypothesis. Indeed, if we exclude items 2, 7 and 9 from the linear regression in Figure 2 in the main text, then the coefficients for individualising arguments and the interaction effect for binding arguments to conservatives increase and become more significant, while the other coefficients remain similarly nonsignificant, and the adjusted R^2 increases from .050 to .071.

	Estimate	Std. err.	t	Lower	Upper
(Intercept)	1.77	1.54	1.15	−1.23	4.76
Opinion	−0.28	0.01	−25.27	−0.31	−0.26
Binding args	1.67	2.19	0.76	−2.60	5.94
Individualising args	6.46	2.17	2.98	2.23	10.70
Conservative	−1.72	0.86	−2.00	−3.41	−0.04
Binding arg × conservative	4.09	1.22	3.37	1.72	6.47
Individualising arg × conservative	0.60	1.21	0.49	−1.77	2.96

Table S3: Linear mixed model of change in opinion for all moral items, clustered by respondent. Coefficients are listed with standard errors, t values, and 95% confidence intervals, computed by likelihood profiles using a likelihood ratio test. The independent variables are initial opinion (−100 to 100); receiving binding or individualising arguments (compared to none); a scale measure for ideology from extremely liberal (−3) through moderate (0) to extremely conservative (3); and interactions between ideology and type of argument received.

D. Linear models

Initial opinions on the nine moral stances were very varied, and could potentially influence attitude change. In our main analysis, we measure average opinion change across the issues (providing the same effect sizes, but with a more straightforward analysis), but to control for initial opinion, the data points need to be separated into the nine moral items for each respondent. This calls for a mixed model, clustered by respondents, who introduce an additional source of variance. The fixed effects are reported in Table S3.

The effects in the main analysis remain when controlling for initial opinion. Initial opinion does have a significant influence on how much the respondents change their opinion: the less they agree initially, the more they move towards the moral stance. Political ideology also has a significant effect (though including a nonsignificant interaction term with initial opinion makes it nonsignificant). As hypothesised, individualising arguments have a significant general effect, while binding arguments has one when given to conservative respondents.

We hypothesise that there should be an even stronger association between opinion change and moral foundation support than ideology. At the end of the survey, the respondents filled in a Moral Foundation Questionnaire. This order of tasks avoided demand characteristics, where the respondents figure out the purpose of the study, which might influence the responses. On the other hand, it runs the risk of posttreatment bias, where conditioning a variable on the treatment makes the groups dissimilar and produces biased estimates of treatment effects, if the covariate is affected by the treatment. In Moral Foundations Theory, it is typically assumed, though not proven, that individual foundation support is relatively stable over time. We checked whether there were any differences in the covariate on the sample level, by comparing MFQ scores across the treatments. There were no significant differences in individualising ($F(2, 364) = 1.2$, $p = .302$, one-way ANOVA) nor binding ($F(2, 367) = 0.13$, $p = .883$) scores. This applies also to the separate foundations (ranging from $F = 0.23$, $p = .793$, for loyalty, to $F = 1.27$, $p = .280$, for fairness). It might be worth cautioning that this of course only fails to

reject the null hypothesis that MFQ scores are uninfluenced by the treatment, it does not prove it.

With this caution in mind, the results of the OLS linear regression including type of arguments, political ideology and individualising and binding MFQ scores are presented in Table S4, alongside the models excluding MFQ scores for comparison. As expected, controlling for moral foundations in the third model, political ideology becomes non-significant, and the model fit increases significantly ($F = 5.42$, $p < .001$).

It can be noted that the negative coefficient for individualising arguments applies to the extreme reference value where the respondents scored 0 on the individualising foundations (only 2% of the whole sample scored below 2 and the mean was 3.5). The other two significant effects are the predicted ones: individualising foundation support is positively associated with opinion change among those receiving individualising arguments, and binding foundation support among those receiving binding arguments.

E. Power analyses

The power, that is, the ability to detect an overall effect (Cohen's f^2), in the linear models with the given number of respondents and assuming that the real overall effect size is the one measured in our data is .96 for the model with dichotomous political ideology, .98 for the one with a scale measure, and 1.0 for the full model with moral foundations.

For the t-tests of the dichotomous data, the measured effect (Cohen's d) varies from $d = .44$ to $d = .60$ in the cases where we hypothesised a difference (arguments vs. no arguments for conservatives, individualising vs. no or binding arguments for liberals, and binding arguments for conservatives vs. liberals). Assuming that these are the real effects, the power of the tests ranges from .63 to .84. With 50 respondents in each group (the average here is 51) and $d = .5$, the power is .70. For the hypothesised null effect, where we expect the null hypothesis to hold, for binding vs. no arguments to liberals, assuming that binding arguments have the same effect size as individualising arguments ($d = .51$), the power is .68. Furthermore, for the case where related work expects a significant difference but our hypothesis does not, for individualising vs. binding arguments to conservatives, assuming that the difference is the same as for liberals ($d = .60$), the power is .88.

F. Excluded respondents

We excluded those respondents who failed attention checks by answering that it was relevant (slightly or above) when assessing the morality of an action whether someone was good at maths or who did not (at least moderately) agree that it is better to do good than bad.

While there were no significant demographic and political differences between the included and excluded respondents (though men were somewhat overrepresented among the excluded ones: there are 45% men in the included sample and 56% in the excluded, $\chi^2(1) = 2.95, p = .086$), the two groups behaved differently when filling in the surveys in such a way as to suggest that the responses from the respondents failing attention checks are indeed of lower quality. For the respondents in the control condition, we have two measurements of the same variable,

	Arguments	Ideology	Moral found.
(Intercept)	−2.43±1.61	−2.42±1.60	0.56±7.95
	(.133)	(.131)	(.944)
Binding args	3.13±2.31	3.07±2.29	9.67±12.53
	(.176)	(.180)	(.441)
Individualising args	7.62±2.28	7.56±2.27	−38.82±12.36
	(<.001)	(<.001)	(.002)
Conservative		−0.22±0.90	−0.06±1.13
		(.806)	(.955)
Binding moral			−0.52±2.07
			(.800)
Individualising moral			−0.53±2.34
			(.820)
Binding args			
× conservative		3.23±1.27	−0.29±1.76
		(.011)	(.869)
× binding moral			7.45±3.26
			(.023)
× individualising moral			−7.24±3.80
			(.057)
Individualising args			
× conservative		−0.00	1.14±1.74
		(.999)	(.512)
× binding moral			2.83±3.06
			(.356)
× individualising moral			11.23±3.46
			(.001)
N	375	375	362
F-statistic	5.63	4.57	4.96
	(.004)	(<.001)	(<.001)
Adjusted R^2	.024	.046	.108

Table S4: Linear models (OLS) of average change in opinion per respondent. Coefficients are listed with standard errors. *P*-values are shown below each coefficient in parentheses. Ideology uses a scale measure from extremely to slightly liberal (−3 to −1) through moderate (0) to slightly to extremely conservative (1 to 3). The independent variables are type of argument received (with no argument as a reference), being conservative (rather than liberal), moral foundation scores, and interactions (denoted by ×) between type of argument and the other variables.

	Arguments	Ideology
(Intercept)	−0.15±1.51	−0.11±1.50
	(.920)	(.944)
Binding args	0.71±2.23	0.55±2.21
	(.749)	(.804)
Individualising args	5.13±2.19	5.05±2.18
	(.020)	(.021)
Conservative		−0.56±0.84
		(.508)
Binding args × conservative		3.39±1.24
		(.006)
Individualising args × conservative		0.39
		(.750)
N	463	460
F-statistic	3.13	3.32
	(.044)	(.006)
Adjusted R^2	.009	.024

Table S5: Linear models (OLS) of average change in opinion per respondent, including all respondents. Coefficients are listed with standard errors. *P*-values are shown below each coefficient in parentheses.

opinion on the moral issues, where we expect no change from attentive respondents, which can thus be used as a response quality check, based on consistency. Checking for reliability between responses, the Intraclass Correlation Coefficient (ICC(1,1)) for included respondents is .75 ($F = 7.12$, $df = 126$, $p < .001$), while for the excluded ones it is only .54 ($F = 3.33$, $df = 41$, $p < .001$). For those who failed the moral relevance control (whether someone was good at maths), it is as low as .46 ($F = 2.71$, $df = 29$, $p = .004$). The two-way mixed effects consistency coefficient (ICC(3,1)) and the Pearson correlation coefficient is .01–.02 larger in all cases. Looking at exceedingly large average changes between the two measurements, 10% of the excluded respondents changed by at least 40 points, while 1.6% of the included ones did the same (where both numbers are halved if the limit is set at 50 points).

We have rerun the main analyses including all respondents. Table S5 lists linear regression results from one model including only the argument treatments and one with ideology added. The results are similar to those of Table 2 in the main manuscript (or the first two columns of Table S4), with the same coefficients being significant. The overall model fit is lower, indicating more variance in the data, which may again point to more noise in the responses from those who failed attention checks.

Table S6 lists linear regression results investigating how MFQ results correspond to opinion change in the three argument treatments. There is one major difference to Table 3 of the main manuscript: the coefficient for the binding foundations score is significantly positive in the individualising argument treatment, which is not predicted here nor by other hypotheses in the related literature. That effect is driven by respondents who answered that it is morally relevant

	Control	Binding	Individualising
(Intercept)	1.75±7.22	5.88±9.53	−30.65±8.81
	(.809)	(.539)	(<.001)
Conservative	−1.19±.98	0.36±1.26	−0.16±1.26
	(.976)	(.773)	(.902)
Binding moral	1.73±1.84	6.78±2.39	4.40±2.17
	(.347)	(.005)	(.044)
Individualising moral	−1.92±2.20	−6.45±2.85	6.99±2.37
	(.384)	(.026)	(.004)
N	165	135	148
F-statistic	.58	6.60	5.77
	(.627)	(<.001)	(<.001)
Adjusted R^2	−.008	.111	.089

Table S6: Linear models (OLS) of average change in opinion per respondent in the three conditions (type of argument received), including all respondents. Coefficients are listed with standard errors. P-values are shown below each coefficient in parentheses.

whether someone is good at maths. A possible explanation is that those respondents tend to answer higher on the scale across all items, and thus their responses would have a greater impact on variables where other respondents tend to score low, such as binding foundations scores in samples that are not exclusively conservative. Indeed, those respondents agree more with the moral issues than the remaining sample (21.1 vs. 12.9; $t(81.8) = 2.51$, $p = .014$, Welch two sample two-sided t-test) and their binding foundations score is larger (3.0 vs. 2.4; $t(87.1) = 5.30$, $p < .001$) without them being more conservative on average (0.22 vs. −0.06; $t(82.2) = 1.17$, $p = .247$).

G. Survey protocol

Pre-screening

What is your age?

What is your gender?

- Male
- Female

Which of the following best describes your highest achieved education level?

- Some High School
- Some college, no degree
- Associate’s degree
- Bachelor’s degree
- Graduate degree (Masters, Doctorate, etc.)

What is your total annual income?

- Don't know/Prefer not to say
- Under $12,500
- $12,500–$24,999
- $25,000–$37,499
- $37,500–$49,999
- $50,000–$62,499
- $62,500–$74,499
- $75,000–$87,499
- $85,000–$99,999
- Over $100,000

How would you describe your political views?

- Don't know/Prefer not to say
- Extremely liberal
- Liberal
- Slightly liberal
- Moderate
- Slightly conservative
- Conservative
- Extremely conservative
- Other

Do you identify with a political group?

- No
- Democrat
- Independent
- Republican
- Other

[New page]

[All respondents who chose Moderate or one of the conservative options and a proportion, selected at random, of those who chose the liberal options in "How would you describe your political views?" were asked:

Do you want to take the full survey on your opinions on current issues? For this you will receive a bonus of $0.30.]

First round

Instructions

You will be presented with nine moral claims. Please select for each statement how much you

agree on a scale from -100 to 100.

[Nine pages, each with the following content. The pages were presented in random order.]

Claim:

{Moral stance X}

How much do you agree with the claim?

[Slider with integer steps from -100 to 100. When pulling the slider, a text next to the slider showed a qualitative interpretation of the number according to the list below.]

- $[-100, -90[$: Disagree extremely strongly
- $[-90, -60[$: Disagree very strongly
- $[-60, -30[$: Disagree strongly
- $[-30, 0[$: Disagree
- $]0, 30]$: Agree
- $]30, 60]$: Agree strongly
- $]60, 90]$: Agree very strongly
- $]90, 100]$: Agree extremely strongly

Second round

Instructions

You will be presented with nine moral claims [treatment: along with arguments for each]. Please [treatment: read the arguments carefully and] select for each claim how much you agree [treatment: with the statement] on a scale from -100 to 100.

[Nine pages, each with the following content. The pages were presented in random order.]

Claim:

{Moral stance X}

[In the binding/individualising treatment:

Argument:

{Binding/individualising argument X}]

How much do you agree with the claim?

[Slider with integer steps from -100 to 100. When pulling the slider, a text next to the slider showed a qualitative interpretation of the number according to the list below.]

- $[-100, -90[$: Disagree extremely strongly
- $[-90, -60[$: Disagree very strongly
- $[-60, -30[$: Disagree strongly
- $[-30, 0[$: Disagree

-]0, 30]: Agree
-]30, 60]: Agree strongly
-]60, 90]: Agree very strongly
-]90, 100]: Agree extremely strongly

[New page]

Instructions

When you decide whether something is right or wrong, to what extent are the following considerations relevant to your thinking?

[The following items were presented in random order.]

Whether or not . . .

- someone suffered emotionally [counted towards harm/care score]
- someone cared for someone weak or vulnerable [harm/care]
- someone was cruel [harm/care]
- some people were treated differently than others [fairness]
- someone acted unfairly [fairness]
- someone was denied his or her rights [fairness]
- someone's action showed love for his or her country [ingroup]
- someone did something to betray his or her group [ingroup]
- someone showed a lack of loyalty [ingroup]
- someone showed a lack of respect for authority [authority]
- someone conformed to the traditions of society [authority]
- an action caused chaos or disorder [authority]
- someone violated standards of purity and decency [purity]
- someone did something disgusting [purity]
- someone acted in a way that God would approve of [purity]
- someone was good at math [control]

[The items and answer options were presented in a table with a column for the items and one column each with radio buttons for the answer options below, allowing exactly one answer per item.]

- not at all relevant [counted as 0 points towards the relevant score]
- not very relevant [1 p]
- slightly relevant [2 p]
- somewhat relevant [3 p]
- very relevant [4 p]
- extremely relevant [5 p]

[New page]

Instructions

Please read the following sentences and indicate your agreement or disagreement.

[The following items were presented in random order.]

- Compassion for those who are suffering is the most crucial virtue [counted towards harm/care score]
- One of the worst things a person could do is hurt a defenseless animal [harm/care]
- It can never be right to kill a human being [harm/care]
- When the government makes laws, the number one principle should be ensuring that everyone is treated fairly [fairness]
- Justice is the most important requirement for a society [fairness]
- I think it's morally wrong that rich children inherit a lot of money while poor children inherit nothing [fairness]
- I am proud of my country's history [ingroup]
- People should be loyal to their family members, even when they have done something wrong [ingroup]
- It is more important to be a team player than to express oneself [ingroup]
- Respect for authority is something all children need to learn [authority]
- Men and women each have different roles to play in society [authority]
- If I were a soldier and disagreed with my commanding officer's orders, I would obey anyway because that is my duty [authority]
- People should not do things that are disgusting, even if no one is harmed [purity]
- I would call some acts wrong on the grounds that they are unnatural [purity]
- Chastity is an important and valuable virtue [purity]
- It is better to do good that to do bad [control]

[The items and answer options were presented in a table with a column for the items and one column each with radio buttons for the answer options below, allowing exactly one answer per item.]

- Strongly disagree [counted as 0 points towards the relevant score]
- Moderately disagree [1 p]
- Slightly disagree [2 p]
- Slightly agree [3 p]
- Moderately agree [4 p]
- Strongly agree [5 p]
